# Dummy run quality assurance study in the Korean Radiation Oncology Group 19 − 09 multi-institutional prospective cohort study of breast cancer

**DOI:** 10.1186/s13014-022-02140-0

**Published:** 2022-11-16

**Authors:** Myeongsoo Kim, Boram Park, Haksoo Kim, Yeon-Joo Kim, Dong Ju Choi, Weonkuu Chung, Yeon Joo Kim, Hyun Soo Shin, Jung Ho Im, Chang-Ok Suh, Jin Hee Kim, Boram Ha, Mi Young Kim, Jongmoo Park, Jeongshim Lee, Sung-Ja Ahn, Sun Young Lee, Grace Kusumawidjaja, Faye Lim, Won Kyung Cho, Haeyoung Kim, Doo Ho Choi, Won Park

**Affiliations:** 1 Department of Radiation Oncology, National Cancer Center, Goyang, Korea; 2 Biomedical Statistics Center, Samsung Medical Center, Seoul, Korea; 3 Radiation Oncology Kyung Hee University Hospital at Gangdong, Kyung Hee University School of Medicine, Seoul, Korea; 4 Department of Radiation Oncology, Kangwon National University Hospital, Chuncheon, Korea; 5 Department of Radiation Oncology, CHA Bundang Medical Center, CHA University, Seongnam, Korea; 6 Department of Radiation Oncology, Keimyung University Dongsan Medical Center, Daegu, Korea; 7 Department of Radiation Oncology, Hallym University Dongtan Sacred Heart Hospital, Hwaseong, Korea; 8 Department of Radiation Oncology, Kyungpook National University Chilgok Hospital, Daegu, Korea; 9 Department of Radiation Oncology, School of Medicine, Kyungpook National University, Daegu, Korea; 10 Department of Radiation Oncology, Inha University Hospital, Incheon, Korea; 11 Department of Radiation Oncology, Chonnam National University Hwasun Hospital, Jeollanam-do Gwangju, Korea; 12 Department of Radiation Oncology, Jeonbuk National University Hospital, Jeonju, Korea; 13 Division of Radiation Oncology, National Cancer Centre Singapore, Singapore, Singapore; 14 Department of Radiation Oncology, Samsung Medical Center, Sungkyunkwan University School of Medicine, Seoul, Korea; 15 Proton Therapy Center, National Cancer Center, 323 Ilsan-ro, Ilsandong-gu, 410-769 Goyang-si, Gyeonggi-do Republic of Korea

**Keywords:** Breast neoplasms, Radiotherapy, Quality assurance, Dummy run, Dosimetric variations

## Abstract

**Background:**

The Korean Radiation Oncology Group (KROG) 19 − 09 prospective cohort study aims to determine the effect of regional nodal irradiation on regional recurrence rates in ypN0 breast cancer patients. Dosimetric variations between radiotherapy (RT) plans of participating institutions may affect the clinical outcome of the study. We performed this study to assess inter-institutional dosimetric variations by dummy run.

**Methods:**

Twelve participating institutions created RT plans for four clinical scenarios using computed tomography images of two dummy cases. Based on a reference structure set, we analyzed dose-volume histograms after collecting the RT plans.

**Results:**

We found variations in dose distribution between institutions, especially in the regional nodal areas. Whole breast and regional nodal irradiation (WBI + RNI) plans had lower inter-institutional agreement and similarity for 95% isodose lines than WBI plans. Fleiss’s kappa values, which were used to measure inter-institutional agreement for the 95% isodose lines, were 0.830 and 0.767 for the large and medium breast WBI plans, respectively, and 0.731 and 0.679 for the large and medium breast WBI + RNI plans, respectively. There were outliers in minimum dose delivered to 95% of the structure (D95%) of axillary level 1 among WBI plans and in D95% of the interpectoral region and axillary level 4 among WBI + RNI plans.

**Conclusion:**

We found inter-institutional and inter-case variations in radiation dose delivered to target volumes and organs at risk. As KROG 19 − 09 is a prospective cohort study, we accepted the dosimetric variation among the different institutions. Actual patient RT plan data should be collected to achieve reliable KROG 19 − 09 study results.

## Background

Regional recurrence is rare, even without regional nodal irradiation (RNI), in T1–3N1 breast cancer patients with ypN0 after neoadjuvant chemotherapy (NAC) and breast-conserving surgery (BCS) [1]. In a retrospective study in Korea of patients with ypN0 after NAC and BCS from 2005 to 2011, only 39.1% received RNI, and it did not improve locoregional control or survival [2]. However, there is no definitive data that support the omission of RNI in patients with ypN0 after NAC and BCS yet, especially in patients who undergo sentinel lymph node biopsy (SLNB).

To determine the effect of RNI on regional recurrence rates in those patients, the Korean Radiation Oncology Group (KROG) initiated a study titled ‘Prospective cohort study to evaluate the effect of regional nodal irradiation omission on the regional recurrence rate after neoadjuvant chemotherapy followed by breast-conserving surgery and sentinel lymph node biopsy in clinical T1-3 with lymph node metastasis in axillary level I and ypN0 breast cancer patient (the KROG 19 − 09 study, CRIS no. KCT0004567)’ in October 2019.

The treatment policy for RNI in ypN0 patients is diverse in Korea. In this prospective cohort study, the institutional policies are respected, and radiation oncologists choose either whole breast irradiation (WBI) or WBI + RNI after discussion with their patients. Radiotherapy (RT) is delivered according to institutional policy.

The KROG 19 − 09 protocol for radiotherapy is as follows: 4–6 megavolt x-ray is recommended; computed tomography (CT) simulation is required; supine position is recommended; either conventional fractionation (daily dose of 1.8-2.0 Gy, 23–28 fractions, total dose of 45-50.4 Gy) or hypofractionation (daily dose of 2.5-3.0 Gy, 13–16 fractions, total dose of 39-43.2 Gy) is allowed; 3-D conformal radiotherapy is recommended; intensity-modulated radiotherapy is allowed; adherence to the ESTRO (European Society for Radiotherapy and Oncology) consensus guideline for clinical target volume (CTV) for whole breast and regional lymph node is recommended; V5Gy, V10Gy, V20Gy, V30Gy, and the mean dose for ipsilateral lung and heart on the dose-volume histogram (DVH) must be recorded. At the kick-off meeting for KROG 19 − 09, we found that the participating institutions use various RT techniques and field designs. It is well known that the actual dose coverage of the regional nodal area varies according to RT technique and field design [3–5]. Variations in dose distribution in the regional nodal area and the organs at risk (OARs) between the participating institutions may affect the clinical outcome of the study. The purpose of this study was to assess inter-institutional dosimetric variations by dummy run.

## Methods

### Dummy run procedure

**Fig. 1:**
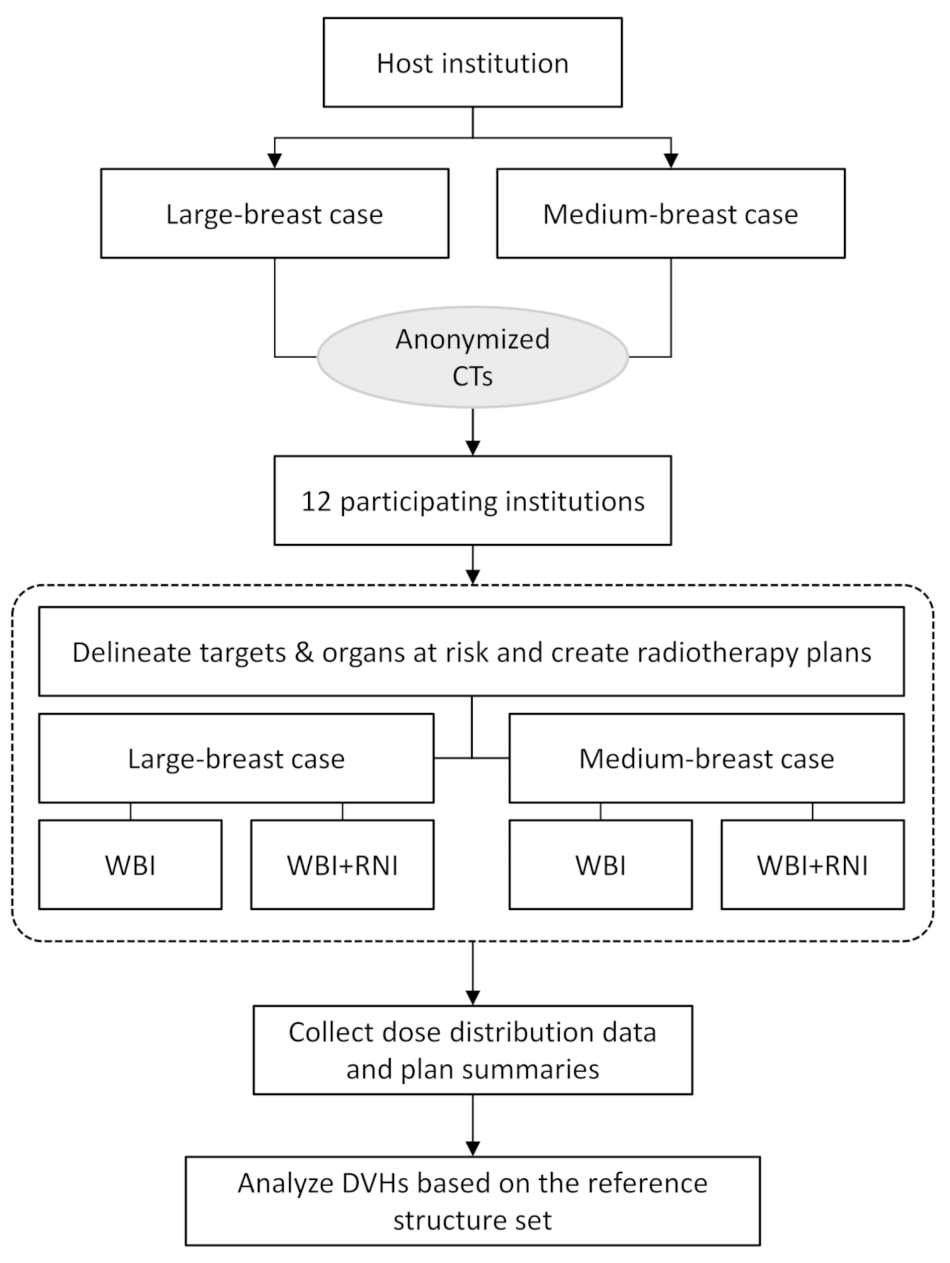
Twelve participating institutions created whole breast irradiation (WBI) and whole breast and regional node irradiation (WBI + RNI) plans using anonymized computed tomography (CT) images of a large- and a medium-breasted case. Dose volume histograms (DVHs) were analyzed based on the reference structure set

Four clinical scenarios, (1) whole breast irradiation (WBI) in a large-breast case, (2) whole breast and regional nodal irradiation (WBI + RNI) in the large-breast case, (3) WBI in a medium-breast case and (4) WBI + RNI in the medium-breast case, were prepared upon which institution-specific radiation treatment plans were created based on each institution’s protocols (Fig. 1). This study does not include information about the patient-specific quality assurance protocols of the treatment plans.

All institutions were also asked to fill out a prepared questionnaire regarding the treatment environment and plan information such as treatment planning system, photon beam energy, dose calculation algorithm, treatment technique, total dose, and fraction number. Anonymized CT images of a large- and a medium-breast case with cT1–3N1 breast cancer on the left side with ypN0 were provided for the treatment planning. In terms of physical characteristics, the large-breast case had a height of 1.57 m, weight of 75 kg, and body mass index (BMI) of 30.4 (kg/m^2^), and the medium-breast case had a height of a 1.61 m, weight of 63 kg and BMI of 24.3 (kg/m^2^).

### Data assessment

The host institution received dose distribution data for the WBI and WBI + RNI plans for the large- and medium-breast cases created by the 12 participating institutions in the format of each institution’s RT Dose Digital Imaging and Communications in Medicine (DICOM) files. Reference CTVs and OARs were delineated according to the ESTRO consensus guideline and a cardiac contouring atlas by a radiation oncologist (Y-J. Kim) [6, 7] to evaluate the DVHs. These reference contours were analyzed by radiation oncology panel reviewers MY. Kim and WK. Cho to ensure they conformed to guidelines. The reference contours were not supplied to the participating institutions but only used for the DVH analysis. The reference structure set included a left breast, interpectoral node CTV (CTVn_intpect), axillae and supraclavicular node CTVs (CTVn_L1-L4), internal mammary node CTV (CTVn_IMN), left lung, heart, shoulder joint with 1 cm margin (sh joint + 1 cm) and left anterior descending coronary artery (LAD_coronary a.).

Based on the reference structure set, we assessed inter-institutional variations in dose to the CTVs and OARs. DVH analysis software developed using ESAPI (eclipse script application pro-gram interface, from Varian’s treatment planning system) was used. For DVH comparisons, we analyzed mean doses and standard deviations for each of the clinical scenarios for all institutions. For CTVs, we calculated the minimum dose delivered to 95% of the structure (D95%). Because of inter-institutional differences in dose fractionation schedules, the results were expressed as D95% as a percentage of the prescribed dose. For the OARs, we calculated the volume receiving 20 Gy (V20Gy) for the left lung; the mean dose and the minimum dose delivered to 5% of the structure (D5%) for the heart and the right breast, respectively; and the volume receiving 20 Gy (V20Gy) and 5 Gy (V5Gy), for the LAD_coronary a. and sh joint + 1 cm, respectively. Boost dose to the tumor bed was not considered when analyzing DVHs. To analyze agreement and similarity of 95% isodose lines between institutions, we utilized two analysis tools; (1) Fleiss’s kappa [8] was calculated using the computational environment for radiotherapy research (CERR) [9] that is MATLAB-based radiotherapy research platform, and (2) Jaccard and Dice similarity coefficients were calculated using MIM (OH, USA) [10, 11]. Fleiss’s kappa value assesses the reliability of agreement between the participating institutions’ isodose lines, which evaluates the degree of inter-variation [8].

### Statistical analysis

Distributions of variables were expressed as medians and ranges (minimum-maximum), and differences between large- and medium-breast cases were assessed using the Wilcoxon rank-sum test. To graphically display the data, we present box plots of five variables: minimum value, maximum value, median, and the first (Q1) and third (Q3) quartiles (i.e., values of the 25th and 75th percentiles of the data set). The interquartile range (IQR) is the distance between the first and third quartiles. If a data value was less than Q1-1.5IQR or greater than Q3 + 1.5IQR, the value was considered an outlier. Outliers were plotted as individual points, and each individual point is labelled with the participating institution and value. A *p*-value less than 0.05 was considered statistically significant. All statistical analysis was performed using SAS software, version 9.4 (SAS Institute Inc., Cary, NC, USA.), and R software, version 4.1.0 (R Project for Statistical Computing).

## Results

### Treatment plan information

As shown in Tables 1, 11 institutions used the Eclipse RTP system (Varian Medical System, Palo Alto, CA), and one used Pinnacle (Philips Healthcare, Andover, MA). Ten institutions used 6MV photon beams for breast and supraclavicular node fields, and two institutions used 6MV beams for breast and 10MV beams for supraclavicular node fields. All institutions calculated dose distributions using an adaptive convolution (AC) (n = 1) or analytical anisotropic algorithm (AAA) (n = 11) to account for inhomogeneity.

**Table 1:** Treatment plan information

Institution	Treatment planning system	Photon beam energy	Dose calculation algorithm	Treatment Technique				Total dose (Gy) / Fractions (no.)			
				**Large breast**		**Medium breast**		**Large breast**		**Medium breast**	
				**WBI**	**WBI** **+RNI**	**WBI**	**WBI** **+RNI**	**WBI**	**WBI** **+RNI**	**WBI**	**WBI** **+RNI**
A	Eclipse v13.7	6MV	AAA	VMAT (2 arcs)	3D (2 fields)	VMAT (2 arcs)	3D (2 fields)	43.2 / 16	43.2 / 16	43.2 / 16	43.2 / 16
B	Pinnacle v9.10	6MV	AC	3D (2 fields)	3D (4 fields)	3D (2 fields)	3D (3 fields)	50 / 25	50 / 25	50 / 25	50 / 25
C	Eclipse v8.9	6MV	AAA	3D (2 fields)	3D (4 fields)	3D (2 fields)	3D (4 fields)	50.4 / 28	50.4 / 28	50.4 / 28	50.4 / 28
D	Eclipse v8.6	6MV	AAA	3D (4 fields)	IMRT (9 fields)	3D (4 fields)	IMRT (10 fields)	41.6 / 16	41.6 / 16	41.6 / 16	41.6 / 16
E	Eclipse v13.7	6MV	AAA	3D (2 fields)	IMRT (4 fields)	3D (2 fields)	IMRT (5 fields)	50 / 25	50 / 25	42.4 / 16	42.4 / 16
F	Eclipse v15.5	6MV	AAA	3D (2 fields)	3D (3 fields)	3D (2 fields)	IMRT (7 fields)	50.4 / 28	50.4 / 28	50.4 / 28	50.4 / 28
G	Eclipse v10	6MV	AAA	3D (2 fields)	3D (4 fields)	3D (2 fields)	3D (4 fields)	50 / 25	50 / 25	50 / 25	50 / 25
H	Eclipse v13.6	6,10MV	AAA	3D (2 fields)	IMRT (6 fields)	3D (2 fields)	IMRT (6 fields)	42.56 / 16	50 / 25	42.56 / 16	50 / 25
I	Eclipse v15.5	6MV	AAA	3D (2 fields)	3D (3 fields)	3D (2 fields)	3D (3 fields)	50 / 25	50 / 25	50 / 25	50 / 25
J	Eclipse v13.0	6MV	AAA	IMRT (6 fields)	IMRT (7 fields)	IMRT (6 fields)	IMRT (6 fields)	50 / 25	50 / 25	50 / 25	50 / 25
K	Eclipse v13.7	6MV	AAA	IMRT (6 fields)	IMRT (8 fields)	IMRT (6 fields)	IMRT (6 fields)	50 / 25	50 / 25	42.7 / 16	42.7 / 16
L	Eclipse v13.6	6,10MV	AAA	3D (2 fields)	3D (4 fields)	3D (2 fields)	IMRT (3 fields)	40.05 / 15	40.05 / 15	40.05 / 15	40.05 / 15

WBI, whole breast irradiation; WBI + RNI, whole breast irradiation plus regional node irradiation; AAA, anisotropic analytic algorithm; VMAT, volume-modulated arc therapy; 3D, three-dimensional conformal radiation therapy AC, adaptive convolution; IMRT, intensity-modulated radiation therapy

For the WBI plans, nine institutions used standard tangential field three-dimensional conformal radiation therapy (3D-CRT) for the large- and medium-breast cases. Three employed intensity-modulated radiation therapy (IMRT) for both cases. Notably, volumetric-modulated arc therapy (VMAT) was used by one institution in the WBI plan of both cases. For the WBI + RNI plans, for the large-breast case, seven institutions used 3D-CRT and five used IMRT, and for the medium-breast case, five used 3D-CRT and seven used IMRT.

The prescription dose (Gy) for all treatment plans of participating institutions ranged from 40.05 to 50.4 Gy, and the fraction numbers ranged from 15 to 28. The prescribed dose per fraction was 1.8 to 2.7 Gy. All the dose fractionation schedules are accepted as biologically equivalent for breast cancer.

### Radiation dose variation between institutions

**Fig. 2:**
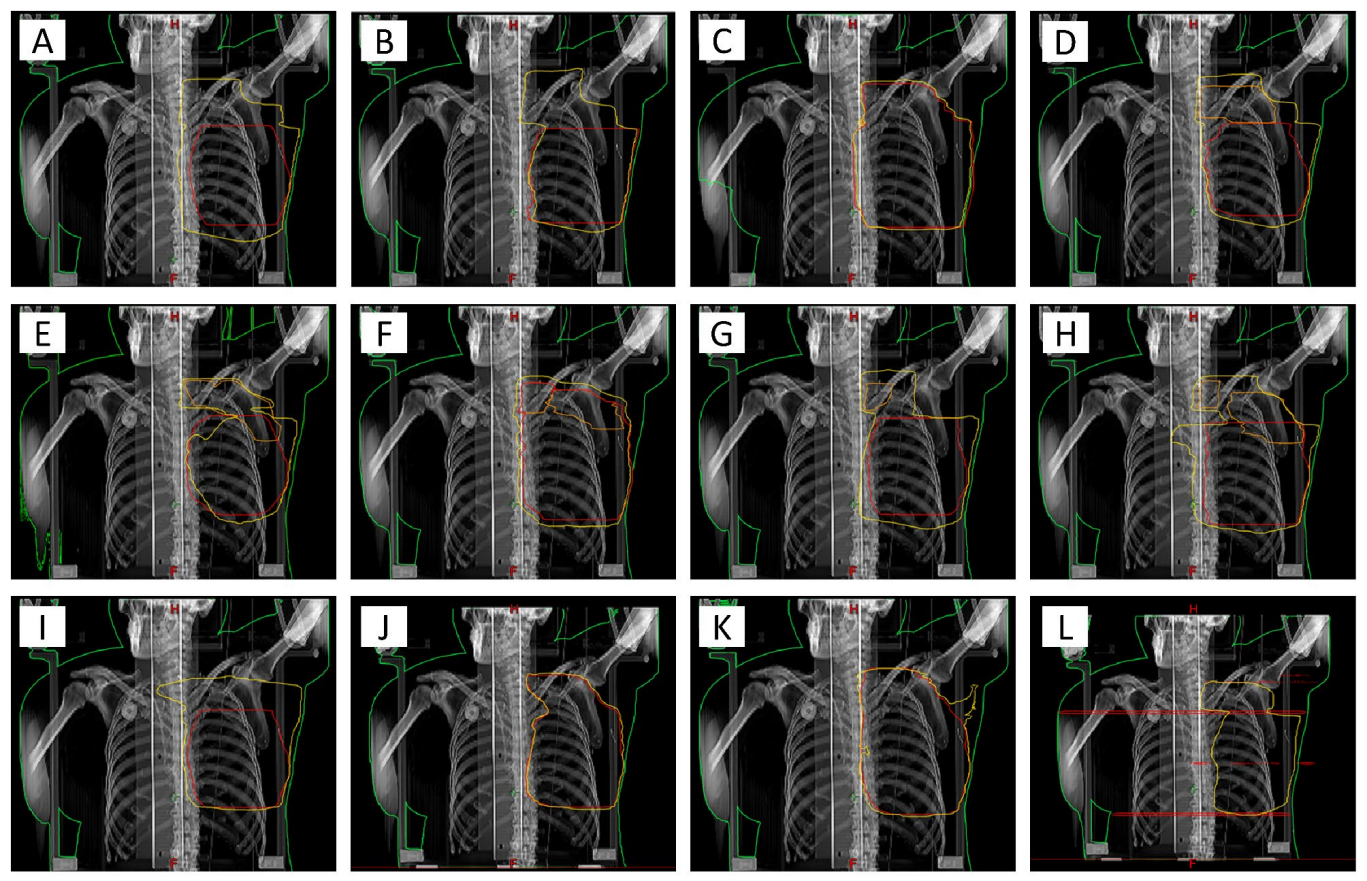
Breast target and regional node target contours (red and orange lines), and 95% isodose lines (yellow line) from the participating institutions for whole breast and regional node irradiation (WBI + RNI) plans in the large-breast case

Figure 2 presents the target contours and isodose lines from the participating institutions for their WBI + RNI plans for the large-breast case. The red and orange lines represent the breast and regional node targets, and the yellow lines are the 95% isodose lines. One institution (L) did not delineate the breast or regional node targets, and three (A, B, I) delineated only breast targets. In these institutions, the RT fields were designed according to anatomical landmarks. We found variations in dose distributions between institutions, especially in the regional nodal areas. As shown in Table 2, Fleiss’s kappa values, which were used to measure inter-institutional agreement for the 95% isodose lines, were 0.830 and 0.767 for the large and medium breast WBI plans, respectively, and 0.731 and 0.679 for the large and medium breast WBI + RNI plans, respectively. The WBI + RNI plans had lower inter-institutional agreement and similarity for 95% isodose lines than the WBI plans.

**Table 2:** Isodose line agreement and similarity

			Large breast		Medium breast	
			**WBI**	**WBI + RNI**	**WBI**	**WBI + RNI**
	**Volume (STD)**		1548.2 (201.2)	2032.0 (379.5)	560.1 (148.7)	906.2 (160.6)
**95% isodose line**	**Fleiss’s kappa**		0.830	0.731	0.767	0.679
	**Jaccard (STD)**		0.766 (0.070)	0.648 (0.053)	0.752 (0.097)	0.602 (0.053)
	**Dice (STD)**		0.866 (0.045)	0.785 (0.040)	0.855 (0.067)	0.751 (0.041)

WBI, whole breast irradiation; WBI + RNI, whole breast irradiation plus regional node irradiation; STD, standard value

**Fig. 3:**
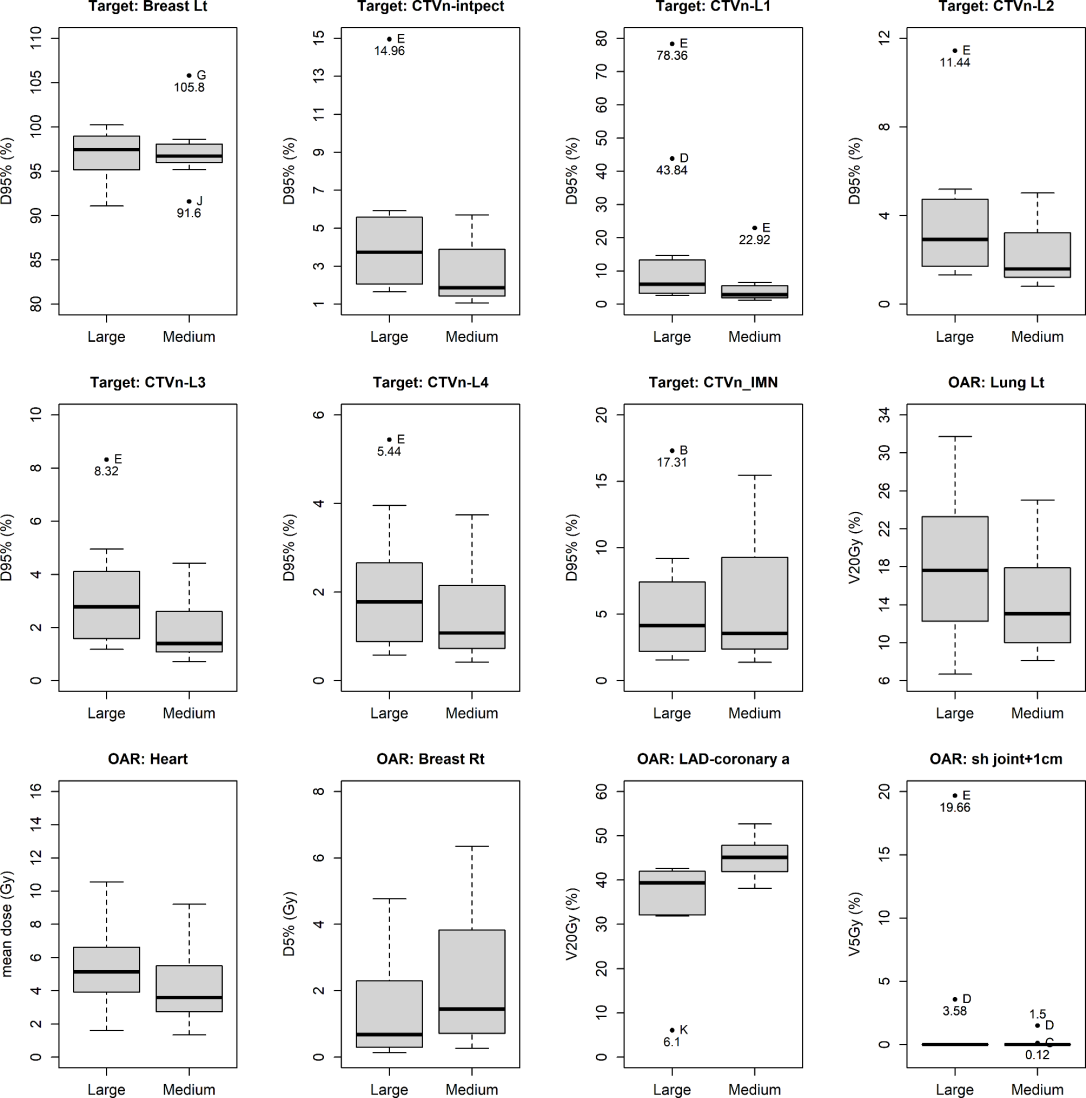
Box plots comparing whole breast irradiation alone plans (Abbreviations: Breast Lt, Breast left; D95%, minimum dose delivered to 95% of the structure; CTVn-intpect, interpectoral node clinical target volume; CTVn-L1-4, axillae level 1–4 clinical target volumes CTVn-IMN, internal mammary node clinical target volume; OAR, organs at risk; Lung Lt, Lung left; V20Gy, volume receiving 20 Gy; Breast Rt, Breast right; D5%, minimum dose delivered to 5% of the structure; LAD_coronary a, left anterior descending coronary artery; sh joint + 1 cm, shoulder joint with 1 cm margin; V5Gy, volume receiving 5 Gy)

**Fig. 4:**
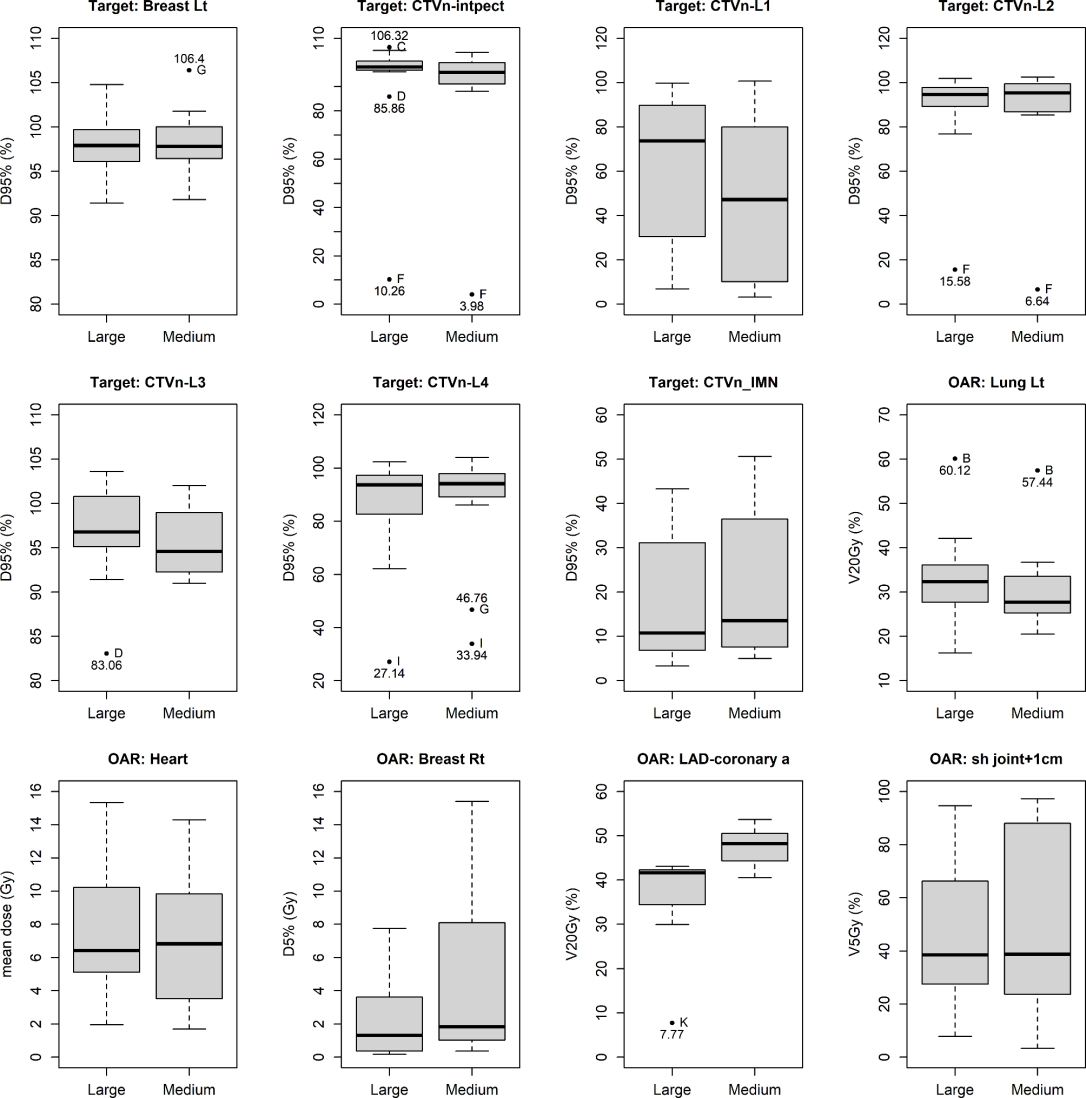
Box plots comparing whole breast irradiation with regional node irradiation plans (Abbreviations: Breast Lt, Breast left; D95%, minimum dose delivered to 95% of the structure; CTVn-intpect, interpectoral node clinical target volume; CTVn-L1-4, axillae level 1–4 clinical target volume; CTVn-IMN, internal mammary node clinical target volume; OAR, organs at risk; Lung Lt, Lung left; V20Gy, volume receiving 20 Gy; Breast Rt, Breast right; D5%, minimum dose delivered to 5% of the structure; LAD_coronary a, left anterior descending coronary artery; sh joint + 1 cm, shoulder joint with 1 cm margin; V5Gy, volume receiving 5 Gy)

There were three outliers among WBI plans with significant differences in D95% of axillary level 1 (Fig. 3). This means that at these institutions, cases received significant doses to the axillary level 1, even with a WBI plan. There were several outliers among WBI + RNI plans with significant differences in D95% of the interpectoral region and axillary level 4 (Fig. 4). This means that cases did not receive a sufficient dose to these areas at these institutions, even with a WBI + RNI plan.

### Radiation dose variation between cases

The differences in dose distributions between the large- and medium-breast treatment plans are shown in Table 3. In the WBI plans, the median D95% (%) of CTVn-L1 for the large- and medium-breast cases were 6.0% (range, 2.6-78.4%) and 2.9% (1.2-22.9%), respectively. This difference was statistically significant (*p*-value = 0.0304). There was a statistically significant difference in LAD_coronary a. V20Gy (%) (*p*-value = 0.0024, median; 39.4% (range, 6.1-42.6%) vs. 45.1% (range, 38.1-52.7%) for the large- and medium-breast cases, respectively). The was also a statistically significant difference in LAD_coronary a. V20Gy (%) in the WBI + RNI plans (*p*-value = 0.0007), median; 41.6% (range, 7.8-43.1%) vs. 48.2% (40.5-53.7%) for large- and medium-breast cases, respectively).

**Table 3:** Comparison between large- and medium-breast cases

			WBI			WBI + RNI		
			**Large breast**	**Medium breast**	***p*** **-value**	**Large breast**	**Medium breast**	***p*** **-value**
Target	Breast Lt	D95% (%)	97.4 (91.1-100.2)	96.7 (91.6-105.8)	0.7508	97.9 (91.4-104.8)	97.8 (91.8-106.4)	0.8399
	CTVn-intpect	D95% (%)	3.7 (1.6–15.0)	1.9 (1.1–5.7)	0.0566	98.2 (10.3-106.3)	96.0 (4.0-104.1)	0.3123
	CTVn-L1	D95% (%)	6.0 (2.6–78.4)	2.9 (1.2–22.9)	0.0304	73.7 (6.9–99.7)	47.2 (3.2-100.6)	0.3708
	CTVn-L2	D95% (%)	2.9 (1.3–11.4)	1.6 (0.8-5.0)	0.0605	94.7 (15.6-101.8)	95.4 (6.6-102.6)	0.7508
	CTVn-L3	D95% (%)	2.8 (1.2–8.3)	1.4 (0.7–4.4)	0.0606	96.8 (83.1-103.6)	94.6 (91.0-102.0)	0.3708
	CTVn-L4	D95% (%)	1.8 (0.6–5.4)	1.1 (0.4–3.7)	0.4704	93.7 (27.1-102.3)	94.1 (33.9–104.0)	0.7508
	CTVn_IMN	D95% (%)	4.1 (1.6–17.3)	3.6 (1.4–15.5)	0.9539	10.8 (3.3–43.3)	13.5 (5-50.6)	0.6236
OAR	Lung Lt	V20Gy (%)	17.6 (6.7–31.7)	13.1 (8.1–25)	0.2602	32.4 (16.3–60.1)	27.7 (20.5–57.4)	0.2602
	Heart	Mean dose (Gy)	5.1 (1.6–10.6)	3.6 (1.3–9.2)	0.1749	6.4 (2.0-15.3)	6.8 (1.7–14.3)	0.7508
	Breast Rt	D5% (Gy)	0.7 (0.1–4.8)	1.4 (0.3–6.4)	0.1332	1.3 (0.2–7.7)	1.8 (0.4–15.4)	0.1841
	LAD-coronary a	V20Gy (%)	39.4 (6.1–42.6)	45.1 (38.1–52.7)	0.0024	41.6 (7.8–43.1)	48.2 (40.5–53.7)	0.0007
	sh joint + 1 cm	V5Gy (%)	0.0 (0.0-19.7)	0.0 (0.0-1.5)	0.8389	38.5 (7.9–94.7)	38.8 (3.3–97.3)	0.977

WBI, whole breast irradiation; WBI + RNI, whole breast irradiation plus regional node irradiation; Breast Lt, Breast left; D95%, minimum dose delivered to 95% of the structure; CTVn-intpect, interpectoral node clinical target volume; CTVn-L1-4, axillae level 1–4 clinical target volume; CTVn-IMN, internal mammary node clinical target volume; OAR, organs at risk; Lung Lt, Lung left; V20Gy, volume receiving 20 Gy; Breast Rt, Breast right; D5%, minimum dose delivered to 5% of the structure; LAD_coronary a, left anterior descending coronary artery; sh joint + 1 cm, shoulder joint with 1 cm margin; V5Gy, volume receiving 5 Gy

## Discussion

In this dummy run study, we found variations in radiation dose delivered to target volumes and organs at risk between institutions. As KROG 19 − 09 is a prospective cohort study, we accepted the dosimetric variation among the different institutions. Although IMRT is available at all the participating institutions, a majority use 3D plans for breast cancer because of limitations in resources. Some institutions still use anatomical landmarks without CTV contouring to reduce workload. The idea that irradiation of the entire lymphatic system may not be necessary for oncologic benefit and the low incidence of severe toxicity with breast RT plans are cited as reasons for using anatomical landmarks without CTV contouring. With a 3D plan, it is inevitable that unintentional doses are delivered to unintended areas. Another limitation of 3D plans is an unavoidable low dose at the field junction. Differences in RT techniques are the main reason for the dose variations between institutions.

As shown in previous international trial dummy runs, it is essential to implement quality assurance to allow the quality of trial data to be optimized and quantified [12–14]. In 2017, a phase III randomized trial was initiated by the Korean Radiation Oncology Group (the KROG 17 − 01 study, NCT03269981) to analyze the impact of RNI in pN1 breast cancer patients receiving effective systemic therapy. The primary objective of the KROG 17 − 01 study was to compare disease-free survival between WBI and WBI + RNI in pN1 breast cancer patients who received BCS and taxane-based chemotherapy. For adequate interpretation of the KROG 17 − 01 study results, an in-silico planning study comparing radiation dose distributions to the regional lymph nodes between the WBI and WBI + RNI plans of institutions participating in the KROG 17 − 01 study was performed [15]. The study found that the relative nodal dose was significantly lower with WBI than WBI + RNI (*p*-value < 0.01) in all nodal regions. It also found moderate-to-strong agreement in radiotherapy treatment volumes between the participants. Significant proportions of radiation were unintentionally delivered to the axillary lymph node level 1 and IMN regions in the WBI plans. Our findings agree with the KROG 17 − 01 in-silico study.

Another dummy run study for quality assurance of a randomized trial on IMN irradiation (the KROG 08 − 06 study) reported that the mean radiation dose to the IMN region was 40–74% of the prescribed dose in their WBI arm [16]. In our study, the median D95% (%) of CTVn-IMN for the large- and medium-breast cases were 4.1% (range, 1.6-17.3%) and 3.6% (range, 1.4-15.5%), respectively, in the WBI plans. Compared to the KROG 08 − 06 dummy run study, the radiation dose to the IMN region in the WBI alone plan was lower in our study.

Recently an insightful dose evaluation study was published [5]. The study reconstructed the treatment plans of the landmark Z0011 [17], AMAROS [18], EORTC 22,922 − 10,925 [19], and MA.20 [20] randomized lymph node irradiation trials to assess the dose distribution to actual lymph node metastases and the ESTRO-CTVs. They searched the study protocols of the Z0011, AMAROS, EORTC, and MA.20 trials for specifications regarding the treatment planning procedure. The field arrangements described in the protocols were used to imitate the 2D treatment plans on 3D computed tomography datasets of (1) a standard patient, (2) an obese patient with large breasts, and (3) a slender patient with small breasts. In these landmark trials, dose distributions at the axillary level 1, 2, 3 and the supraclavicular and IMN regions varied. These variations resulted from differences in RT techniques and field designs in each trial. They also found that the extent of incidental irradiation to the axillary nodes depended clearly on the patient’s body shape. In line with this previous study, we found inter-institutional and inter-case dose variations.

Variations in dose distribution at the regional nodal areas and OARs between participating institutions may affect the KROG 19 − 09 study’s clinical outcomes. To reduce the dosimetric variation among institutions, we plan to provide feedback from periodic audits. Furthermore, actual patient RT plan data should be collected to analyze the effect of these variations on regional recurrence rates and toxicity. To ensure reliable results, participants of KROG 19 − 09 agreed to the collection of actual patient RT plan data. We plan to collect DVH data by using artificial intelligence (AI)-based auto contouring software to delineate CTV and OAR structures. We will be able to analyze the relationship between the radiation dose and recurrence or toxicity rates based on the segmented structures with these data.

There are several limitations to this study. First, we performed the dummy run study in only two different breast-sized cases because of resource limitations. The inclusion of more cases with more diverse breast sizes could have provided more information about dose variation. Second, some institutions dropped out of and others joined KROG 19 − 09 after this dummy run study. Therefore, the results of this dummy run study are not able to reflect all the participants of KROG 19 − 09. However, this study is worthwhile as it allowed the participants to agree on collecting actual patient RT planning data in order to obtain reliable results for KROG 19 − 09.

In conclusion, in this dummy run study, we found inter-institutional and inter-case variations in radiation dose delivered to target volumes and organs at risk. As KROG 19 − 09 is a prospective cohort study, we accepted the dosimetric variation among the different institutions. Actual patient RT plan data should be collected to achieve reliable KROG 19 − 09 study results.

## Data Availability

The supporting data is available.
